# SIRT1 protects rat lung tissue against severe burn-induced remote ALI by attenuating the apoptosis of PMVECs via p38 MAPK signaling

**DOI:** 10.1038/srep10277

**Published:** 2015-05-20

**Authors:** Xiaozhi Bai, Lei Fan, Ting He, Wenbin Jia, Longlong Yang, Jun Zhang, Yang Liu, Jihong Shi, Linlin Su, Dahai Hu

**Affiliations:** 1 Department of Burns and Cutaneous Surgery, Xijing Hospital, Fourth Military Medical University, Xi’an, Shaanxi, China; 2 Department of Burn and Plastic Surgery, No.205 Hospital of Chinese People’s Liberation Army, Jinzhou, Liaoning, China

## Abstract

Silent information regulator type-1 (SIRT1) has been reported to be involved in the cardiopulmonary protection. However, its role in the pathogenesis of burn-induced remote acute lung injury (ALI) is currently unknown. The present study aims to investigate the role of SIRT1 in burn-induced remote ALI and the involved signaling pathway. We observed that SIRT1 expression in rat lung tissue after burn injury appeared an increasing trend after a short period of suppression. The upregulation of SIRT1 stimulated by resveratrol exhibited remission of histopathologic changes, reduction of cell apoptosis, and downregulation of pro-inflammatory cytokines in rat pulmonary tissues suffering from severe burn. We next used primary pulmonary microvascular endothelial cells (PMVECs) challenged by burn serum (BS) to simulate *in vivo* rat lung tissue after burn injury, and found that BS significantly suppressed SIRT1 expression, increased cell apoptosis, and activated p38 MAPK signaling. The use of resveratrol reversed these effects, while knockdown of SIRT1 by shRNA further augmented BS-induced increase of cell apoptosis and activation of p38 MAPK. Taken together, these results indicate that SIRT1 might protect lung tissue against burn-induced remote ALI by attenuating PMVEC apoptosis via p38 MAPK signaling, suggesting its potential therapeutic effects on the treatment of ALI.

A severe burn injury often leads to systemic inflammatory response syndrome (SIRS), sepsis, multiple organ dysfunction syndrome (MODS) and acute lung injury (ALI)/acute respiratory distress syndrome (ARDS) which are common causes of morbidity and mortality^1^. ALI is a leading complication in patients with extensive deep burns in which burned area exceeds 30% of the total body surface area (TBSA)^2^. Although the pathophysiologic mechanism underlying burn-induced ALI remains incompletely elucidated, growing evidences from experimental and clinical studies have shown that both the systemic inflammatory response and oxidative stress play central roles in the development of ALI^3^,^4^,^5^. Endothelial cell (EC) injury may also play a critical role in the occurrence and progression of ALI. Previous studies have demonstrated that inflammation and further damages, including the apoptosis of ECs, are considered as essential early steps of ALI, while the endothelial dysfunction and pulmonary microvascular hyper-permeability to fluids and proteins are the hallmarks of ALI and sepsis^6^,^7^. Thus, preventing endothelial injury has been developed as a potential therapeutical strategy for treating ALI.

Sirtuins, the class III nicotinamide adenine dinucleotide (NAD^+^)-dependent deacetylases, are emerging regulators of various biological processes^8^. In mammals, seven sirtuins have been described^9^, most studies have focused on silent information regulator type-1 (SIRT1) whereas the other six sirtuins have been less investigated. SIRT1 has been shown to play a role in transcriptional and post-transcriptional regulation of gene expression through the deacetylation of histone and non-histone proteins^8^. Recent data have suggested that SIRT1 targets p53^10^, Ku70^11^ and the forkhead transcription factors^12^ for deacetylation, and thus regulates stress responses, apoptosis and cellular senescence^13^.

Resveratrol (3,5,4-trihydroxystilbene) has been shown to activate SIRT1^14^ and exhibit various bioactivities^15^, including anti-oxidative^16^, anti-tumorigenic^17^, anti-angiogenic^18^, anti-inflammatory^19^, and neuroprotective^20^ effects. Recent studies have shown that resveratrol leads to the amelioration of staphylococcal enterotoxin B-induced lung injury^21^ and attenuates the apoptosis of pulmonary microvascular endothelial cells (PMVECs) induced by high shear stress and pro-inflammatory factors^22^. However, the underlying mechanism is largely unclear. Here we hypothesized that the activation of SIRT1 by resveratrol might protect ECs against burn-induced ALI.

In the present study, we have shown that resveratrol exerted protective roles in rat lung after severe burn injury by modulating SIRT1 expression. In addition, our results have demonstrated that the protective effect of resveratrol on lung tissue might be through attenuating the inflammatory damage and PMVEC apoptosis by inhibiting p38 mitogen activated protein kinase (MAPK) pathway.

## Results

### Both the mRNA and protein levels of SIRT1 in rat lung tissue increase over time after burn injury

**Figure 1:**
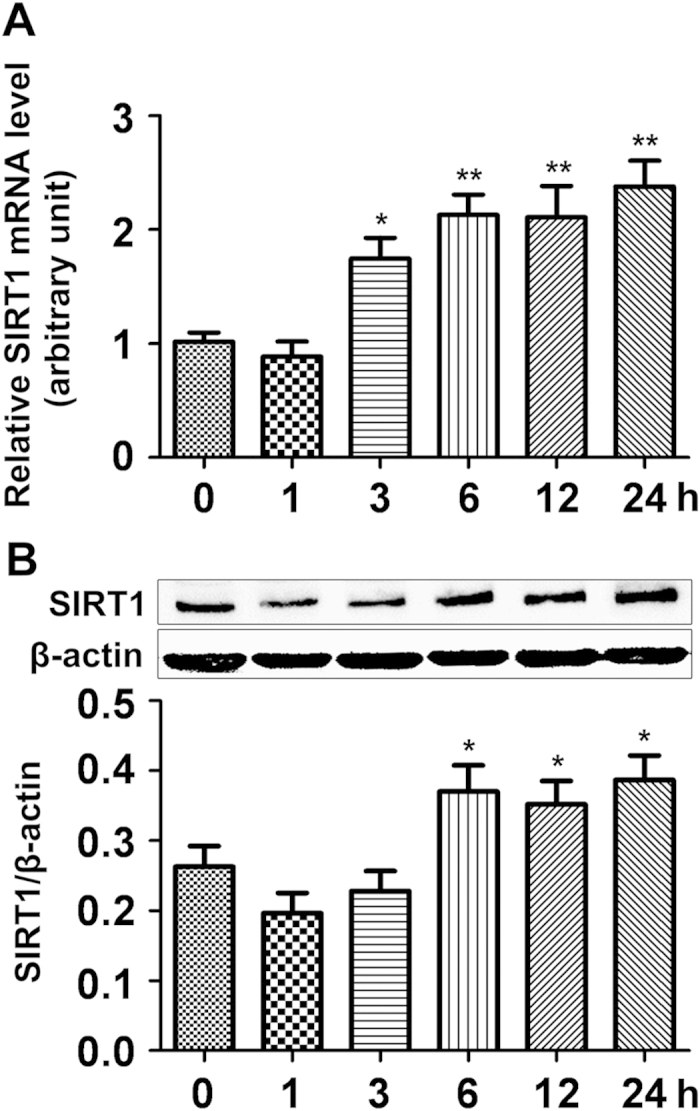
The expression of SIRT1 in lung tissue from severely burned rats over time. **** (**A**) The mRNA level of SIRT1 in rat lung after burn injury was analyzed by real-time PCR and normalized against *GAPDH* mRNA level. (**B**) Representative immunoblots showing the protein level change of SIRT1 in the lung of severely burned rats over time. Results represent mean ± SEM of six independent experiments using different rats. *p < 0.05; **p < 0.01, compared to the value at 0h.

To understand whether burn injury would affect SIRT1 expression in the lung, we first conducted qRT-PCR and Western blot to assess SIRT1 expression at both mRNA and protein levels. Results showed that SIRT1 mRNA level was slightly suppressed at the early stage at 1 h post-burn and then significantly increased by approximately 2-fold at 3 h and lasted till 24 h post-burn (Fig. 1A); SIRT1 protein level showed the same trend except that the early suppressive effect appeared at both 1 h and 3 h post-burn, and the upregulation of SIRT1 began at 6 h and lasted till 24 h post-burn injury (Fig. 1B).

### Resveratrol recovers the histopathologic changes in rat lung tissue induced by burn injury

**Figure 2:**
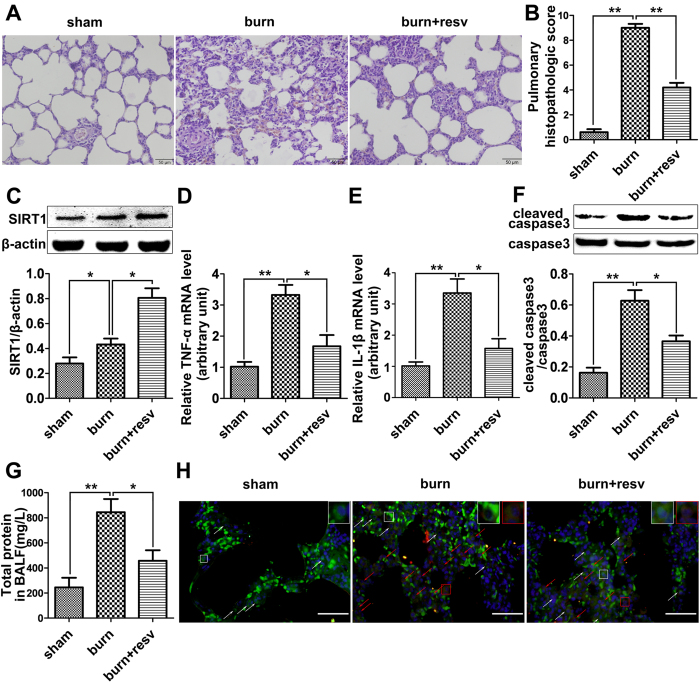
*In vivo* studies to assess the effects of resveratrol in rat lung with burn-induced ALI. (**A**) H&E staining showing the morphological changes of the lung tissue from rats receiving severe burn injury at 24 h post-burn. The burn group (burn) showed increasing lung edema, alveolar hemorrhage, neutrophil infiltration, and destroyed epithelial/endothelial cell structure (*middle panel*) compared to the sham burn group (sham*, left panel*), while the resveratrol-treated burn group (burn + resv) showed significant improvement on lung morphology (*right panel*). Scale bar = 50 μm. (**B**) The pulmonary histologic score was counted at 24 h post-burn, and resveratrol significantly attenuated burn-induced high histologic score. (**C**) The protein level of SIRT1 was further significantly elevated by its activator resveratrol in burned rat lung. (**D**) Real-time PCR analysis showing the effect of resveratrol on the mRNA level of TNF-α in burned rat lung at 12 h post-treatment. (**E**) Real-time PCR analysis showing the effect of resveratrol on the mRNA level of IL-1β in burned rat lung at 12 h post-treatment. (**F**) Representative immunoblots showing the effect of resveratrol on the cleaved caspase-3 level in burned rat lung at 12 h post-treatment. (**G**) Total protein contents in BALF were measured in each of the three groups, and resveratrol significantly reversed the increased protein content induced by severe burn at 12 h post-treatment. (**H**) PMVECs positively stained with both CD31 (*green*) and cleaved caspase-3 (*red*) represent the apoptotic cells (Red arrows direct to apoptotic ECs while white arrows direct to normal ECs. Scale bar = 50 μm). Results represent mean ± SEM of six independent experiments using different rats. *p < 0.05; **p < 0.01, compared to the value in the burn group.

We next moved to assess the effect of resveratrol, a known SIRT1 activator, on the morphological changes of rat lung tissue after burn injury by hematoxylin and eosin (H&E) staining. The sham burn group (Fig. 2A, *left panel*) showed the same normal morphology as that in normal rat lung tissue. The lung tissue from rats receiving burn treatment was sectioned and stained 24 h post-injury, it showed remarkable pro-inflammatory alterations characterized by lung edema, alveolar hemorrhage, neutrophil infiltration, and destruction of epithelial and endothelial cell sheets (Fig. 2A, *middle panel*). In contrast in resveratrol-treated burn group, multiple features of ALI such as neutrophil infiltration, interstitial edema, and pulmonary hemorrhage showed notable improvement (Fig. 2A, *right panel*). The pulmonary histopathologic scores from above three treatment groups were evaluated by Gloor double blind score system^23^. Score in the burn group significantly increased by almost 13 folds, while this effect was attenuated by resveratrol administration (Fig. 2B).

### Resveratrol further enhances SIRT1 expression, while recovers the levels of TNF-a, IL-1b and cleaved caspase-3 induced by burn injury in rat lung

As resveratrol is a known SIRT1 activator, we first examined SIRT1 level after resveratrol treatment in the lung tissue from burned rats. Results showed that resveratrol further enhanced the elevated level of SIRT1 induced by burn injury (Fig. 2C). It is known that inflammatory mediators play an important role in the pathogenesis of severe burn-induced remote ALI. Thus we next examined the mRNA levels of two inflammatory cytokines, TNF-α and IL-1β, in lung tissue after burn injury by qRT-PCR. Results showed that both the mRNA levels of TNF-α (Fig. 2D) and IL-1β (Fig. 2E) were significantly upregulated by 3 folds when measured at 12 h post-injury, however, this effect was remarkably attenuated by resveratrol treatment (Fig. 2D-E). In addition, we examined the level of cleaved caspase-3, the active form of caspase-3, to investigate if apoptosis occurred in this study. Results showed that the protein level of cleaved caspase-3 was significantly increased when measured at 12 h post-burn injury, while it was reduced by resveratrol treatment (Fig. 2F).

### Resveratrol reduces the permeability of pulmonary microvascular endothelium and maintains its integrity

To investigate whether resveratrol could reduce the leakage of proteins into bronchoalveolar and thus protect the lung, we next moved to measure the protein content in the bronchoalveolar lavage fluid (BALF). Results showed that the protein content in BALF significantly increased at 12 h post-burn, while resveratrol remarkably reversed this induction at 12 h post-treatment (Fig. 2G), suggesting that the integrity of pulmonary microvascular endothelium destroyed by burn injury could be improved by resveratrol treatment. To clarify whether the apoptosis of PMVECs could decrease by resveratrol intervene, lung tissue sections were double-stained with anti-CD31 (a putative PMVEC marker, *green*) and anti-cleaved caspase-3 (*red*). Immunofluorescence results showed that cleaved caspase-3 seldom expressed in the sham group, while the number of cells positively stained with both CD31 and cleaved caspase-3 in burn group was much more than that in the sham group. Importantly, resveratrol significantly reduced the expression of cleaved caspase-3 in cells targeted by CD31 antibody (Fig. 2H).

### Resveratrol protects PMVECs against burn serum-induced apoptosis *in vitro*

**Figure 3:**
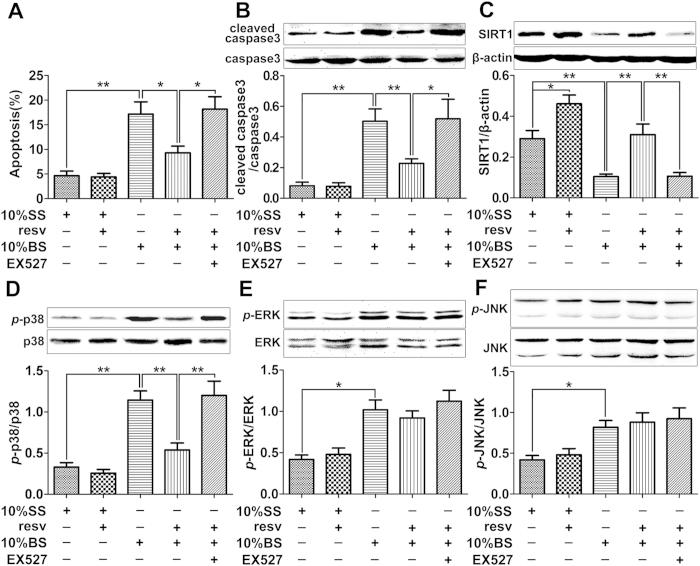
Effects of resveratrol and/or EX527 on cell apoptosis and MAPK signaling in cultured PMVECs challenged with burn serum. **** PMVECs were exposed to 10% sham serum (SS), 10% SS + resveratrol (resv) (20 μM), 10% burn serum (BS), 10% BS + resv, or 10% BS + resv + EX527 (1 μM) for 12 h. (**A**) Flow cytometry analysis showing the percentage of apoptotic cells in each treatment group. (**B**) Representative immunoblots showing the protein level change of cleaved caspase-3 in each treatment group. (**C**) Representative immunoblots showing the protein level change of SIRT1 in each treatment group. (**D**-**F**) Representative immunoblots showing the protein level changes of *p*-p38 (**D**), *p*-ERK (**E**), and *p*-JNK (**F**) in each treatment group. Results represent mean ± SEM of six independent experiments using different batches of PMVECs from different rats. *p < 0.05; **p < 0.01, compared to the value in 10% BS group or 10% BS + resv group.

We further moved to the *in vitro* study to explore the underlying mechanism regulating SIRT1’s protective role in lung tissue after burn injury. PMVECs were exposed to 10% sham serum (SS) or 10% burn serum (BS) added with or without 20 μM resveratrol (resv) for 12 h, and then cell apoptosis was examined by flow cytometry. Results showed that BS significantly induced cell apoptosis in PMVECs by 3.5-fold, while this effect was remarkably attenuated by administration with resveratrol (Fig. 3A). In addition, the protein level of cleaved caspase-3 was significantly elevated after BS stimulation, while this effect was reversed by resveratrol, and the co-administration of resveratrol and EX527, a SIRT1 inhibitor, again upregulated the level of cleaved caspase-3 (Fig. 3B).

### The effects of SIRT1 activator and/or inhibitor on the activation of three major MAPKs in cultured PMVECs stimulated by burn serum

PMVECs were exposed to 10%SS, 10% SS + resv, 10% BS, 10% BS + resv, or 10% BS + resv + EX527 for 12 h, then the expression levels of SIRT1 and three major forms of MAPKs, p38, ERK and JNK, were examined by Western blots. Results showed that SIRT1 level was significantly suppressed by BS compared to that in SS group, while resveratrol effectively reversed SIRT1 level’s decrease (Fig. 3C). Results also showed that the protein level of phosphorylated p38 (*p*-p38) was significantly elevated after BS treatment, while this effect was reversed by resveratrol, and the co-administration of resveratrol and EX527 again upregulated *p*-p38 level (Fig. 3D). Although BS also induced the upregulation of phosphorylated ERK (*p*-ERK) (Fig. 3E) and phosphorylated JNK (*p*-JNK) (Fig. 3F) levels, neither resveratrol nor resveratrol + EX527 showed any effects on further protein level changes.

### Knockdown of SIRT1 in PMVECs by shRNA further augments cell apoptosis induced by burn serum via the p38 MAPK signaling

**Figure 4:**
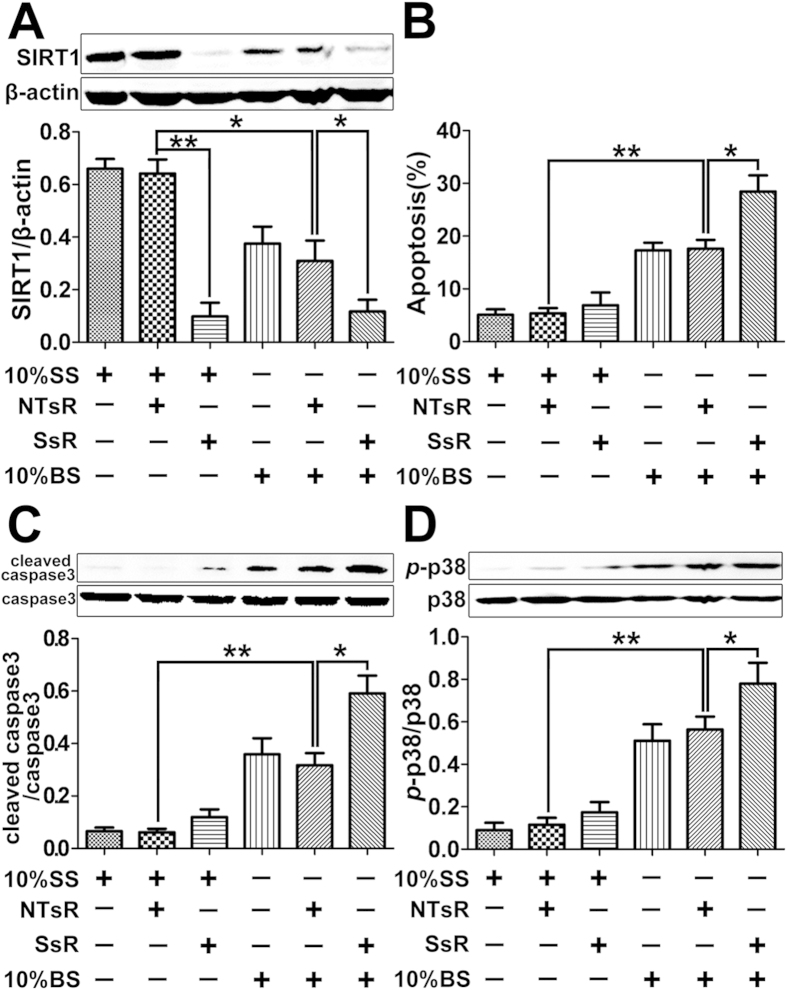
Effects of SIRT1 knockdown by shRNA on cell apoptosis and p38 MAPK signaling in cultured PMVECs challenged with burn serum. **** PMVECs were exposed to 10% sham serum (SS), 10% SS + non-targeting shRNA (NTsR), 10% SS + SIRT1 shRNA (SsR), 10% burn serum (BS), 10% BS + NTsR, 10% BS + SsR for 12 h. (**A**) Representative immunoblots showing the protein level change of SIRT1 in each treatment group. (**B**) Flow cytometry analysis showing the percentage of apoptotic cells in each treatment group. (**C**) Representative immunoblots showing the protein level change of cleaved caspase-3 in each treatment group. (**D**) Representative immunoblots showing the protein level changes of *p*-p38 in each treatment group. Results represent mean ± SEM of six independent experiments using different batches of PMVECs from different rats. *p < 0.05; **p < 0.01, compared to the value in 10% BS + NTsR group.

Knockdown of SIRT1 in PMVECs by shRNA was performed to further confirm the anti-apoptosis effect of SIRT1 after burn injury. PMVECs were treated with 10% SS, 10% SS + non-targeting shRNA (NTsR), 10% SS + SIRT1 shRNA (SsR), 10% BS, 10% BS + NTsR, or 10% BS + SsR for 12 h, the efficiency of SIRT1 knockdown in PMVECs by shRNA was first examined by Western blot. Results showed that SIRT1 shRNA significantly decreased SIRT1 protein level in both SS and BS groups (Fig. 4A), indicating the effectiveness of the SIRT1 shRNA used. Cell apoptosis was then examined by flow cytometry and results showed that knockdown of SIRT1 by shRNA further significantly augmented PMVECs apoptosis induced by BS stimulation (Fig. 4B). In addition, SIRT1 knockdown further upregulated the level of cleaved caspase-3 induced by BS (Fig. 4C). We next moved to further confirm whether p38 MAPK signaling was involved in the inhibitory effects of SIRT1 on BS-induced apoptosis. Results showed that knockdown of SIRT1 by shRNA further activated p38 MAPK induced by BS (Fig. 4D). These results indicated that the inhibition of SIRT1 expression could aggravate cell apoptosis via the activation of p38 MAPK pathway.

### Resveratrol attenuates the apoptosis of PMVECs via p38 MAPK signaling

**Figure 5:**
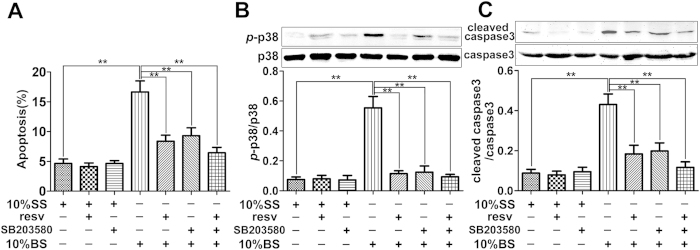
Effects of p38 inhibition on the protective role of resveratrol on PMVECs. **** PMVECs were exposed to 10% sham serum (SS), 10% SS + resveratrol (resv) (20 μM), 10% SS + SB203580 (20 μM), 10% burn serum (BS), 10% BS + resv, 10% BS + SB203580, 10% BS + resv + SB203580 for 12 h. (**A**) Flow cytometry analysis showing the percentage of apoptotic cells in each treatment group. (**B**) Representative immunoblots showing the protein level changes of *p*-p38 in each treatment group. (**C**) Representative immunoblots showing the protein level change of cleaved caspase-3 in each treatment group. Results represent mean ± SEM of six independent experiments using different batches of PMVECs from different rats. *p < 0.05; **p < 0.01, compared to the value in 10% BS group.

PMVECs were treated with 10% SS, 10% SS + resv, 10% SS + SB203580, 10% BS, 10% BS + resv, 10% BS + SB203580, or 10% BS + resv + SB203580 for 12 h. Results showed that the use of SB203580 (a specific inhibitor of p38) alone or with resv significantly attenuated the apoptosis of PMVECs (Fig. 5A), downregulated the phosphorylation of p38 (Fig. 5B), and reduced the level of cleaved caspase-3 after BS stimulation (Fig. 5C).

## Discussion

ALI and its extreme manifestation ARDS, are the well-documented major causes of morbidity and mortality in burned patients admitted to the hospital, especially in patients with combined smoke inhalation injury or delayed resuscitation^2^,^24^,^25^. Pulmonary pathology in major thermal injury is found in 30~80% of burn fatalities^26^. Immediately after severe burn, hypovolemic shock develops and the generalized inflammatory cascade is initiated^27^,^28^. Edema in lung tissue is the most rapid in the first 6 to 24 h after burn^28^,^29^. Within 48 h after burn, the body usually remains infection-free; however, virtually all cell-mediated and humoral immune functions become deranged^27^. Thus, the lung is frequently being the first organ to fail, even in the absence of inhalational injury^29^. Consistent with prior work defining burn-induced ALI, our lung injury model involving a 30% TBSA full-thickness burn, resulted in a dramatic increase in pulmonary edema, neutrophil infiltration and histologic changes (Fig. 2A-B). Meanwhile, the increased protein content in BALF indicates an increase of the permeability of pulmonary microvascular endothelium and an impairment of its integrity (Fig. 2G). Also, ECs apoptosis increased during this period suggests the remote pulmonary injury after severe burn (Fig. 2H).

Identifying early mediators in burn-induced ALI and the underlying molecular mechanism is of critical importance while it is not fully understood yet. Growing evidences from experimental and clinical studies have shown that endothelial dysfunction and pulmonary microvascular hyper-permeability play a central role in the development of ALI^6^,^7^. The pulmonary endothelium is a continuous monolayer of squamous cells that internally lines blood vessels. It is together with the collagen-rich basement membrane and separates but also selectively connects tissue microenvironment and blood to help maintain tissue homeostasis^30^. The integrity of microvascular endothelium prevents the extensive leakage of large molecular from serum, and the excessive deposition of large molecular like proteins in the alveolar often leads to infection, anhelation and dysfunction of oxygenation. The present research showed that the protein content in BALF increased significantly after severe burn without inhalation injury, indicating the integrity of pulmonary microvascular endothelium was impaired as PMVECs were injured (Fig. 2G). ALI is associated with an intense pulmonary inflammatory response with the accumulation of both pro- and anti-inflammatory mediators^31^. When the pro-inflammatory process dominates, the endothelial injury often leads to alterations in metabolic functions which contribute to ARDS pathogenesis^32^. In our model, we demonstrated that a significant increase of pro-inflammatory mediators, such as TNF-α (Fig. 2D) and IL-1β (Fig. 2E), was detected after burn injury by quantitative real-time PCR analysis.

There are more and more evidences implicating increased epithelial/endothelial cell apoptosis in the pathogenesis of ALI. Studies in critically ill patients have shown that ALI is associated with increased cell death rate^33^,^34^, whereas the investigation of the effect of apoptosis inhibitors have shown remarkable increase in cell survival rate using *in vivo* models of sepsis and ALI^35^,^36^,^37^. Caspase-3 plays a key role in the apoptotic cell death^38^, and our analysis revealed a significant increase in the cleaved caspase-3 level after burn injury indicating the enhanced cell apoptosis in pulmonary (Fig. 2F). PMVECs play a key role in maintaining normal oxygenation in pulmonary tissue, thus the extensive apoptosis often leads to the impairment of pulmonary function. To clarify the apoptosis of PMVECs induced by severe burn, immunofluorescence staining was performed. As in Fig. 2H, PMVECs co-stained with CD31 and cleaved caspase-3 showed orange which indicates the apoptotic cells. In accordance with our hypothesis, the apoptosis of PMVECs increased after severe burns. Then we utilized a previously established burn serum-induced PMVEC culture model to investigate the burn serum-induced EC damage. Our results confirmed that challenge of rat PMVECs with 10% serum collected from rats receiving a 30% TBSA burn upregulated the cleaved caspase-3 level (Fig. 3B), and enhanced cell apoptosis detected by flow cytometry (Fig. 3A).

SIRT1 is a NAD^ + ^-dependent class III protein deacetylase involved in cell growth, differentiation, stress resistance, reduction of oxidative damage, and metabolism^8^,^39^,^40^,^41^. The present study is the first to demonstrate that both the mRNA and protein levels of SIRT1 in rat lung were suppressed during the early stage of 1~3 h post burn injury, and then significantly upregulated at later time points (Fig. 1A-B). In contrast, SIRT1 protein level showed remarkable decrease following burn serum stimulation in rat PMVECs (Fig. 3C). Studies have shown that Nicotine and LPS could upregulate both the mRNA and protein levels of SIRT1 in human gingival fibroblasts^42^, also LPS and heat stress could synergistically increase SIRT1 expression in human dental pulp cells^40^. However, Du G *et al.* found that SIRT1 protein expression was reduced by TNF-α in a time- and dose-dependent manner, also TNF-α caused a notable increase in the percentage of apoptotic endothelial progenitor cells^43^. In the present study, the different responses of SIRT1 to burn injury among animal model, cell culture model and previous studies could be explained by the complexity of pulmonary tissue, different cell types involved, and the distinct stimulation used in these trials. The internal environment is in serious disorder after severe burn, which leads to a complicated stimulation to the lung even without inhalation injury. We found that SIRT1 level was transiently inhibited in the very early stage post-burn although there was no statistical difference. The inhibition might be concerned with systemic disorder of the body since organs directly or indirectly affected by burn injury would undergo a complex process to reach a new but transient balance. There is no consensus on how the expression level of SIRT1 changes after stress. It is thought that along with the impairment of the body, the expression of SIRT1 increases through a negative feedback loop to relieve pulmonary inflammation, promote the DNA repair and inhibit the extensive apoptosis of cells. The regulation process is so complicated that further researches are strongly needed to elucidate the regulatory mechanism of strong stress on SIRT1.

The pro-inflammatory cytokine TNF-α induces the production of other inflammatory cytokines, and stimulates the migration and adherence of neutrophils to endothelial cells^44^. Recent studies have shown that SIRT1 took anti-inflammatory effects in primary human coronary artery endothelial cells^45^ and human airway smooth muscle cells^46^. In the current study, the activation of SIRT1 by resveratrol reversed the increase of TNF-α and IL-1β levels in lung tissues from rats with burn-induced ALI (Fig. 2D-E), indicating that resveratrol could attenuate burn-induced lung injury by decreasing TNF-α and IL-1β expression. This result was further confirmed by the improvement on neutrophil infiltration, interstitial edema, and pulmonary hemorrhage in the lung after resveratrol administration as shown in Fig. 2A.

Resveratrol is known as a putative activator of SIRT1^14^, it could prevent cigarette smoke extract-induced apoptosis in ECs^45^. The therapeutic effects of resveratrol on oxidative-stress-induced senescence in ECs could be significantly abolished by SIRT1 knockdown^47^. Thus in this study we hypothesized that resveratrol might protect ECs against apoptosis by the activation of SIRT1 and thus attenuate burn-induced ALI. Results showed that resveratrol reduced pulmonary apoptosis induced by burn injury along with the decreased level of cleaved caspase-3 (Fig. 2F). In accordance with above results, immunofluorescence double-staining showed that the ratio of cleaved caspase-3 positive PMVECs significantly reduced, suggesting that resveratrol could at least partially reverse PMVECs apoptosis induced by the severe burn (Fig. 2H).

To further confirm the observed protective role of SIRT1 in lung tissue from burned rats, the activation of caspase-3 and cell apoptosis were investigated in PMVECs challenged by burn serum at the presence of resveratrol and/or SIRT1 inhibitor EX527. As expected, burn serum strongly increased the apoptosis of PMVECs, which was significantly restored upon resveratrol treatment (Fig. 3A-B). In addition, this restored effect was abolished by SIRT1 inhibitor EX527 (Fig. 3A-B). We further knockdowned SIRT1 by shRNA in PMVECs and found that the apoptosis of PMVECs remarkably increased after burn serum stimulation (Fig. 4A-B). Taken together, our results indicated that the activation of SIRT1 could attenuate the apoptosis of PMVECs and thus alleviate ALI induced by severe burn injury, while the inhibition of SIRT1 could aggravate aforementioned cell apoptosis and burn injury.

SIRT1 has been shown to take effects on anti-inflammation, anti-oxidation, inhibition of DNA damage and attenuation of apoptosis in various cell types^8^,^48^. On the molecular level, several signaling pathways may contribute to the pulmonary protection by SIRT1 activation. SIRT1 overexpression blocked LPS- and nicotine-induced phosphatidylinositol 3-kinase(PI3K), protein kinase C (PKC), p38, ERK, JNK and nuclear factor kappa B (NF-κB) activation^40^,^42^,^49^. To probe the potential relationship between SIRT1 and MAPK signaling in burn-induced apoptosis of PMVECs, we analyzed caspase-3 activity, cell apoptosis and MAPK activation in the presence of specific SIRT1 inhibitor and/or activator. The presence of resveratrol triggered a reduction in apoptotic cell rate (Fig. 3A), caspase-3 activity (Fig. 3B), and p38 activity (Fig. 3D) after burn serum stimulation. However, the co-presence of resveratrol and EX527 reversed above decrease. Interestingly, ERK and JNK signaling were not affected by resveratrol or resveratrol/EX527 co-treatment (Fig. 3E-F). To further confirm the involvement of p38 MAPK in SIRT1-induced protective role in lung tissues from burned rats, PMVECs were transfected with non-targeting shRNA or specific SIRT1 shRNA followed by burn serum stimulation. Results showed that the p38 MAPK and caspase-3 activities in SIRT1 knockdown group further remarkably increased after burn serum stimulation (Fig. 4C-D). When SB203580, a p38 inhibitor, was used to inhibit p38 phosphorylation, similar effects were observed with the use of resveratrol, that is the increased apoptosis of PMVECs induced by burn serum was significantly reduced (Fig. 5A-C). Thus it is reasonable to assume that the protective effect of resveratrol is closely related to the inhibition of p38 MAPK signaling. Some studies have indicated that inhibition of p38 MAPK signaling attenuated apoptosis in human umbilical vein endothelial cells^50^,^51^, suggesting a prominent role of p38 MAPK in SIRT1’s protective role against apoptotic cell death. However, the precise mechanism by which SIRT1 modulates p38 activity needs further elucidation, and this will be addressed in further studies.

In conclusion, this study is the first to demonstrate that SIRT1 activation exhibits protective and anti-apoptotic roles against severe burn-induced ALI possibly through the p38 MAPK pathway. These results suggest that SIRT1 activation might be a potential therapeutic strategy for organ protection after severe burn injury.

## Materials and Methods

### Ethics Statement

All animal experiments were performed in accordance with the guidelines from the Administration of Animal Experiments for Medical Research Purposes issued by the Ministry of Health of China. The protocol used was reviewed and approved by the Animal Experiment Administration Committee of the Fourth Military Medical University (FMMU, Xi’an, China). All procedures were performed under sodium pentobarbital anesthesia. All efforts were made to minimize the suffering of rats during experiments.

### Animal

Ninety-six healthy adult male Sprague-Dawley (SD) rats weighing 200~250 g were included in this study. Animals were provided by Experimental Animal Center of FMMU. Rats were fed *ad libitum* a standard diet and water throughout the study. All animals were housed separately and kept under standard conditions at room temperature (22~24 °C) in a 12 h light/ 12 h dark cycle. Sixty-six rats were used for tissue sampling, twenty for burn serum isolation and ten for PMVEC culture.

### Burn Procedure and Drug Administration *in vivo*

Sixty-six rats were randomly divided into three groups: sham burn (sham, *n* = 6), burn/Ringer’s lactate (burn, *n* = 30), and burn/resveratrol treatment (burn + resv, *n* = 30). In this study we have used a well-established animal burn injury model to systemically induce lung injury^52^,^53^. In brief, SD rats were anesthetized, shaved to remove the fur on the dorsal and lateral back, placed in a mold corresponding to 30% of the TBSA, and then subjected to a full-thickness burn injury by exposure to 92 °C water for 18 sec. Resveratrol (Sigma, St. Louis, MO) was first dissolved in Dimethyl sulfoxide (DMSO) to a concentration of 200 mg/ml, and then diluted in Ringer’s lactate solution yielding a stock concentration of 1 mg/ml. Immediately post-burn, burn + resv group was injected intraperitoneally with diluted resveratrol solution at 50 mg/kg b.w., while the burn group was treated with Ringer’s lactate solution + DMSO (0.5% vol/vol, 50 ml/kg b.w) in the same way. The sham group was subjected to an identical preparation except for immersion in room temperature water. All rats received analgesia (buprenorphine, 0.05 mg/kg b.w. ip) every 8 hours post-burn. Rats were closely monitored during the first 8 hours post-burn to ensure the complete recovery from anesthesia, the disappearance of pain, and the ability to consume food and water. Subsequently, rats in each treatment group were sacrificed at 1, 3, 6, 12 and 24 h post-injury for tissue sampling. Each tissue sample was divided into three parts for Western blot, quantitative real-time PCR and histological examination, respectively.

### Burn Serum Isolation

Twenty rats were randomly divided into two groups: sham burn (sham, *n* = 10) and burn/Ringer’s lactate (burn, *n* = 10). Rats in these two groups were processed in the same way as described above. Burn serum was then collected at 12 h following the burn procedure.

### Histological Examination

Lung specimens were fixed in 10% formalin, dehydrated in alcohol, embedded in paraffin, and then cut into 5-μm thickness sections and mounted. Sections were stained with H&E after deparaffinization as previously described^53^. Histologic changes were graded by two blinded examiners as reported earlier^54^. Lung tissues were scored for intra-alveolar edema, intra-alveolar hemorrhage, and neutrophil infiltration from 0 to 4 (0, absent; 1, mild; 2, moderate; 3, severe; 4, overwhelming) for a maximum score of 12 as described previously by Gloor et al^23^.

### Protein content in BALF detection

SD rats were sacrificed at 12 h post-injury and the lung was extracted. The right side of the lung was ligated while the left-inferior was lavaged twice by cold saline 4 ml per time. The 8 ml of lavage fluid was recyled and half of it was used to lavage the left-inferior twice. The lavage fluid was centrifuged to sediment cells at 300 × g. The the protein content of acellular supernatant was detected by bicinchoninic acid protein assay kit (Pierce, Rockford, IL).

### Immunohistofluorescence

Lung specimens were fixed in 10% formalin, dehydrated in alcohol, embedded in paraffin, and then cut into 5-μm thickness sections and mounted. Sections were deparaffinized with xylene, and heat-mediated Ag retrieval was performed. All of the sections were incubated overnight at 4 °C with anti-CD31 (Abcam, Cambridge, UK) and anti-cleaved caspase-3 (Abcam) primary antibody. On the next day, slides were incubated with anti-mouse Alexa Fluor 488 and anti-rabbit Alexa Fluor 555 (Life, Eugene, OR) secondary antibody at 37 °C for 1 hour. DAPI was used for nuclear staining. Images were analyzed by Image-Pro Plus 6.0 system (Media Cybernetics, Silver Spring, MD).

### PMVECs Culture

The isolation and culture of primary rat PMVECs were performed according to the published methods with minor modification^55^,^56^. Briefly, the fresh lung was aseptically removed and rinsed in PBS twice. After the pleura and the outer edge of lung lobe were cut off, the specimens at 1.5-mm^3^ obtained from the lung surface were carefully placed into tissue culture dishes containing DMEM supplemented with 20% fetal calf serum, 100 U/ml of penicillin–streptomycin, and 25 μg/ml of endothelial cell growth supplement (Gibco, Grand Island, NY) in a 5% CO_2_ incubator at 37 °C. Sixty hours later, residual lung tissues were removed and the culture medium was replaced every three days. When a contact-inhibited monolayer was achieved approximately 1~2 weeks post-plating, cells were passaged with 0.25% trypsin solution. Experimental data were obtained from cells between passages third to fifth.

### shRNA of SIRT1

The recombinant lentivirus vector for SIRT1 silencing (SIRT1-shRNA-GFP) and the negative control lentivirus vector containing non-targeting (NT) shRNA (NT-shRNA-GFP) were purchased from Shanghai Gene Chemistry Company (Shanghai, China). All vectors were labeled with GFP, which served as a detecting marker. PMVECs were transfected with either SIRT1 shRNA or NT shRNA for 48 hours. The efficiency of shRNA transfection was measured by Western blot analysis.

### Drug Administration in PMVECs

For detecting SIRT1 expression and the activities of caspase-3 and MAPKs, approximately 2.0 × 10^6^ serum-starved PMVECs were seeded into 21-cm^2^ dishes, treated with 10% (vol/vol in culture medium) sham serum (SS), 10% SS + resv (20 μM), 10% SS + SB203580 (20 μM) (Sigma, St. Louis, MO), 10% burn serum (BS), 10% BS + resv, 10% BS + resv + EX527 (1 μM) (Sigma, St. Louis, MO), or 10% BS + resv + SB203580 for 12 h. For SIRT1 silencing, PMVECs at the same density as above were transfected with either SIRT1 shRNA or NT shRNA for 48 h and then stimulated by 10% SS or 10% BS for 12 h. Cells were then collected for Western blotting, quantitative real-time PCR and flow cytometry analysis. EX527 was first dissolved in DMSO to 1 mM and then diluted to the final concentration (1 μM) with DMEM (the final DMSO concentration equals 0.1%).

### Western Blotting Analysis

To assess protein levels of SIRT1, cleaved caspase-3, *p*-ERK, *p*-p38 and *p*-JNK after drug treatment or silencing experiment, 50 μg of total protein were subjected to SDS-PAGE and transferred onto PVDF membranes. Membranes were blocked with 5% non-fat milk at room temperature for 3 h, incubated with primary antibodies specific to SIRT1 (1:1000, Abcam, Cambridge, UK), cleaved caspase-3 (1:1000, Abcam), caspase-3 (1:1000, Abcam), *p*-p38 (1:1000, Cell Signaling Technology, Beverly, MA), p38 (1:1000, Cell Signaling Technology), *p*-ERK (1:1000, Cell Signaling Technology), ERK (1:1000, Cell Signaling Technology), *p*-JNK (1:1000, Cell Signaling Technology), JNK (1:1000, Cell Signaling Technology), or β-actin (1:1000, Abcam) at 4 °C overnight. On the next day, membranes were incubated with HRP-conjugated secondary antibodies diluted at 1:3000 (Boster, Wuhan, China) at 37 °C for 1 h. Protein bands on the membrane were visualized with ECL Kit (Millipore, USA) using FluorChem FC system (Alpha Innotech). Results were presented as densitometric ratio between the protein of interest and the loading control (β-actin).

### Quantitative real-time PCR (qRT-PCR)

500 ng of total RNA in the lung tissue from burned rats were extracted using RNA-Isolation Kit (Takara, Japan) and reverse-transcribed using Prime Script™ RT Reagent Kit (Takara). The obtained cDNAs were then amplified with SYBR^®^Premix Ex Taq™ Kit (Takara) with primer pairs specific to each gene (see Supplementary Table S1 online) using Bio-Rad IQ5 Real-Time system (Bio-Rad, Hercules, CA). PCR conditions were as follows: initial denaturation at 95 °C for 30 s, denaturation at 95 °C for 30 s, annealing at 60 °C for 10 s, and elongation at 72 °C for 15 s for total 40 cycles. Results were from six different lung tissues to determine the relative RNA level of each gene, which was normalized against the RNA level of *GAPDH*.

### Flow Cytometry

Apoptosis was detected by Annexin V-FITC Apoptosis Detection Kit I (BD Biosciences, San Diego, CA) following the manufacturer’s instruction. Rat PMVECs were exposed to 10% SS, 10% SS + resv, 10% BS, 10% BS + resv, or 10% BS + resv + EX527 for 12 h. In silencing experiment, cells were pre-transfected with SIRT1 shRNA or NT shRNA for 48 h followed by serum stimulation for 12 h. Cells were then harvested using 0.25% trypsin, washed twice with cold PBS, resuspended in binding buffer, and then incubated with Annexin V-FITC/PI (Merck, Germany) for 15 min at room temperature in dark. Samples were then analyzed by FACS Calibur (BD, USA). The percentage of stained cells was quantified using Cell Quest software (BD FACSDiva Software). Experiments were repeated six times using different batches of cells.

### Statistical Analysis

All data were presented as mean ± SEM. Comparisons between different groups were carried out using one-way analysis of variance (ANOVA) followed by Bonferroni *t* test. Data were analyzed with the SPSS 13.0 program (IBM, Armonk, USA). *p* < 0.05 was accepted as statistically significant.

## Additional Information

**How to cite this article**: Bai, X. *et al*. SIRT1 protects rat lung tissue against severe burn-induced remote ALI by attenuating the apoptosis of PMVECs via p38 MAPK signaling. *Sci. Rep.* **5**, 10277; doi: 10.1038/srep10277 (2015).

## Supplementary Material

Supplementary Information
