# Supplementary material for: SIRT1 protects rat lung tissue against severe burn-induced remote ALI by attenuating the apoptosis of PMVECs via p38 MAPK signaling
Source: Sci Rep. 2015 May 20;5:10277. doi: 10.1038/srep10277 (PMC4445725; doi:10.1038/srep10277)
Supplement: Supplementary Information [file srep10277-s1.pdf]

SITR1 protects rat lung tissue against severe burn-induced remote ALI by attenuating the apoptosis of PMVECs via p38 MAPK signaling

Xiaozhi Bai[1*]; Lei Fan [1*]; Ting He[1*]; Wenbin Jia[1]; Longlong Yang[1]; Jun Zhang[2]; Yang Liu[1]; Jihong Shi[1]; Linlin Su[1#] & Dahai Hu[1#]

[1] Department of Burns and Cutaneous Surgery, Xijing Hospital, Fourth Military Medical University, Xi'an, Shaanxi, China. [2] Department of Burn and Plastic Surgery, No. 205 Hospital of Chinese People's Liberation Army, Jinzhou, Liaoning, China.

[*] These authors contributed equally to this work.

[#]**Corresponding authors**: Dahai Hu, Linlin Su

Address: Department of Burns and Cutaneous Surgery, Xijing Hospital, Fourth Military Medical University, No.127 Changle West Road, Xi'an, 710032, China.

Tel:+86-29-8477 5298

Fax: +86-29-83251734

E-mail address: burnlab@fmmu.edu.cn or linlinsu@fmmu.edu.cn

Reprint requests: Dahai Hu

Address: Department of Burns and Cutaneous Surgery, Xijing Hospital, Fourth Military Medical University, No.127 Changle West Road, Xi'an, 710032, China.

E-mail address: burnlab@fmmu.edu.cn

Table S1. Primer pairs used for qRT-PCR in this study.

Gene	Forward Sequence	Reverse Sequence
SIRT1	5’- CAGACCCTCAAGCCATGTTTGATA -3’	5’- TTGGATTCCTGCAACCTGCTC-3’
TNF-α	5’- GTTCTATGGCCCAGACCCTCAC -3’	5’- GGCACCACTAGTTGGTTGTCTTTG -3’
IL-1β	5’- TCCAGGATGAGGACATGAGCAC -3’	5’- GAACGTCACACACCAGCAGGTTA -3’
GAPDH	5’- TGTGTCCGTCGTGGATCTGA -3’	5’-TTGCTGTTGAAGTCGCAGGAG -3’
